# Advanced Techniques in the Percutaneous Ablation of Liver Tumours

**DOI:** 10.3390/diagnostics11040585

**Published:** 2021-03-24

**Authors:** Terrence CH Hui, Justin Kwan, Uei Pua

**Affiliations:** 1 Department of Diagnostic Radiology, Tan Tock Seng Hospital, Singapore 308433, Singapore; terrencehui123@gmail.com (T.C.H.); justin_kwan@ttsh.com.sg (J.K.); 2 Lee Kong Chian School of Medicine, National Technological University, Singapore 636921, Singapore; 3 Yong Loo Lin School of Medicine, National University of Singapore, Singapore 117597, Singapore

**Keywords:** percutaneous ablation, hepatocellular carcinoma, liver metastases

## Abstract

Percutaneous ablation is an accepted treatment modality for primary hepatocellular carcinoma (HCC) and liver metastases. The goal of curative ablation is to cause the necrosis of all tumour cells with an adequate margin, akin to surgical resection, while minimising local damage to non-target tissue. Aside from the ablative modality, the proceduralist must decide the most appropriate imaging modality for visualising the tumour and monitoring the ablation zone. The proceduralist may also employ protective measures to minimise injury to non-target organs. This review article discusses the important considerations an interventionalist needs to consider when performing the percutaneous ablation of liver tumours. It covers the different ablative modalities, image guidance, and protective techniques, with an emphasis on new and advanced ablative modalities and adjunctive techniques to optimise results and achieve satisfactory ablation margins.

## 1. Introduction

Percutaneous ablation is an accepted treatment modality for primary hepatocellular carcinoma (HCC) and liver metastases [1]. The widely adopted Barcelona clinic liver cancer (BCLC) guidelines for HCC recommend percutaneous ablation as an alternative to surgery for patients with stage 0 (very early stage, single lesion <2 cm) and stage A (early stage, single lesion >2 cm or multifocal disease ≤3 lesions each <3 cm) disease [2,3]. Percutaneous ablation is safe and nearly as efficacious as surgery, usually requiring a lower cost and shorter hospital stay for single HCC <2 cm [4,5,6,7]. Percutaneous ablation has also been proven to achieve a survival benefit in patients with oligometastatic liver disease, with up to nine hepatic metastases [8,9,10,11,12].

Today, percutaneous ablation modalities include radiofrequency ablation (RFA), microwave ablation (MWA), cryoablation, irreversible electroporation (IRE), laser, and high intensity focused ultrasound (HIFU) to name a few. In addition to ablation modality, the proceduralist has to decide the most appropriate imaging modality for guidance, based on tumour visibility and location. The proceduralist must also decide whether to employ various protective techniques to avoid damage to surrounding structures and organs. Percutaneous ablation can be used alone or in combination with other modalities. For example, combination therapy with thermal ablation and trans-arterial chemoembolization is recommended for HCCs larger than 3 cm [13].

The goal of curative ablation is to cause the necrosis of all tumour cells with an adequate margin, akin to surgical resection, while minimising local damage to non-target tissue. Failure to obtain an adequate margin increases the risk of local tumour progression (LTP), a known predictor of poor outcome [14]. A safety margin of 5 mm for HCC is recommended, largely based upon the accepted surgical margin for HCC; this has been shown to be an independent predictor of LTP [15,16]. All hepatic metastases require a 360 degree 1 cm cuff of tumour-free margin, although a 1 cm margin has also been recommended for larger HCC because larger HCCs are more likely to be biologically aggressive and have microsatellites and microvascular invasion not visible on imaging [17,18,19,20].

This review article discusses the important factors which an interventionalist needs to consider when performing the percutaneous ablation of liver tumours. It covers the different ablative modalities, image guidance, and protective techniques with an emphasis on new and advanced ablative modalities and adjunctive techniques to optimise results and achieve satisfactory ablation margins. This article does not discuss (i) the management of antiplatelet/anticoagulation drug therapy; (ii) pain control; or (iii) imaging follow-up.

## 2. Ablative Modalities

Ablative modalities are divided into non-energy ablation (i.e., chemical ablation) and energy-based ablation. Energy-based ablative modalities can further be divided into thermal and non-thermal ablative modalities [21]. This article uses the terminology advocated by Ahmed et al. for image-guided tumour ablation [21]. For instance, the term “applicator” is used when speaking in general about a “needle” device, while specific terms are used when describing a particular ablative modality; for example, “electrodes” are used for RF and IRE applicators and “cryoprobes” are used for cryoablation [21]. 

Alcohol ablation (AA) is a non-energy, chemical ablative modality which induces coagulative necrosis by causing cellular dehydration and protein denaturation. Ethanol is injected directly into the tumour under image guidance, while the surrounding cirrhotic liver limits the diffusion into and around the surrounding liver. AA is commonly used for HCC and is not recommended for hepatic metastasis due to the presence of surrounding normal liver parenchyma, which causes the ethanol distribution to be unpredictable. AA has a high rate of LTP, requiring repeated treatment due to residual tumour cells, and as such, has largely been replaced by energy-based ablation for most lesions [22,23,24,25,26]. AA is reserved for difficult-to-treat tumour locations as monotherapy or in combination, where the use of energy-based modalities alone risks injury to vulnerable structures such as the gallbladder [27,28].

Radiofrequency ablation (RFA) is the first thermal ablation modality to be described and achieves complete ablation in 90% of very early-stage lesions (<2 cm) with a local recurrence rate of 1% [29]. Resistive tissue heating is caused around the RFA electrode as an alternating current is conducted through it, acting as the cathode of a closed electrical circuit, with grounding pads applied to the skin acting as the anode. This heat is conducted through indirect heating of the adjacent tissue, forming the ablation zone via coagulative necrosis. The main limitations of RFA are tissue charring, causing an increase in impedance and peripheral tissue cooling, caused by the dissipation of heat by both large blood vessels as well as capillary level micro-perfusion [30]. Although effective against small (<2 cm) HCCs, achieving adequate margins for larger tumours requires multiple electrodes [22,31]. Due to this limitation, RFA suffers from a relatively high rate of LTP ranging from 10.0 to 39.1% at five years [16,22]. Factors associated with LTP are a larger tumour size >2 cm, tumour without encapsulation, poorly differentiated HCC, subcapsular location, and suboptimal ablative margin adjacent to a nearby vessel, owing to the heat sink effect [32]. 

Microwave ablation (MWA) is a newer technology which uses electromagnetic energy to create an ellipsoidal zone of tissue heating caused by dielectric hysteresis, a process where water molecules are forced to align with an oscillating electric field, creating kinetic energy that is converted to heat. Compared with RFA, MWA is more predictable and produces higher temperatures and larger ablation zones with similar complication rates, without the risk of skin burns from grounding pads [33,34]. By directly heating a zone of tissue, MWA is less reliant on heat conduction, and therefore less susceptible to the heat sink effect [35]. MWA achieves larger ablations and faster ablation times, requiring fewer applicator insertions compared with RFA [36,37]. MWA overcomes the size limitations of RFA and is able to treat hepatic lesions up to 8 cm in size [38]. Additionally, when two or more applicators are used, RF electrodes cannot be “on” simultaneously due to technological limitations, while up to three MWA antennae can be used simultaneously. Regarding the data comparing LTP rates of MWA and RFA, MWA has similar therapeutic effects as RFA with a shorter ablation time for early stage HCC [33,39]. In their meta-analysis, Glassberg et al. found that the use of MWA significantly reduced the LTP rate by 30%, especially for larger tumours (>2.5 cm) [40]. On the other hand, Ding et al. found RFA to have lower LTP rates compared with MWA (5.2% versus 10.9%, respectively) but attributed this difference to the larger size of the tumours being ablated with MWA, an independent predictor of LTP [41].

Laser ablation (LA) is a thermal ablation modality which uses laser optical fibres to deliver high-energy laser radiation to the target tissue leading to coagulative necrosis. The LA applicators are smaller compared with other modalities (22 G) and laser energy is less tissue-sensitive compared with RF. The ablation zone produced is small due to light scattering and reduced energy penetration due to tissue carbonisation; therefore, multiple applicators are often needed to achieve complete ablation [42]. Two trials have shown LA to be as effective and safe as RFA, but it has not been widely adopted, likely due to the popularity of MWA and other ablative modalities [43,44]. 

Cryoablation (CA) uses a combination of rapid cooling followed by thawing leading to coagulative necrosis and cell death by the formation of intracellular ice crystals which interrupts cellular metabolism and causes ischemia via vascular thrombosis. The main advantage of cryoablation over heat-based thermal ablation modalities is the visualisation of the ice ball on computed tomography (CT) (and magnetic resonance imaging (MRI)) and, to a lesser extent, ultrasound (US) [45]. Cryoablation is also less painful compared with RFA and MWA when the ablation zone is anticipated to involve the subcutaneous tissues, pleura, and diaphragm [46]. Better visualisation of the ice ball increases the chance of a complete ablation and reduces the risk of injury to critical structures [47,48]. However, the main drawback and reason why liver cryoablation is not widely adopted is due to an increased risk of bleeding and the added risk of cryoshock [49,50]. Cryoshock is a rare, life-threatening condition that presents with multi-organ failure, severe coagulopathy, and disseminated intravascular coagulation. The pathogenesis related to cryoshock stems from the fact that the ablation zone is re-perfused after the ice ball melts, resulting in the release of cellular debris into the systemic circulation. This life-threatening condition was described to occur in 1% of patients who underwent open or laparoscopic hepatic cryosurgery [51]. However, the exact rate of cryoshock in percutaneous cryoablation remains unknown. Rong et al. did not encounter a single case of cryoshock in a case series of 1401 cryoablations on 866 patients, although reported seven patients (0.6%) who experienced cryoreaction during cryoablation, which manifests as chills, fever, tachycardia, tachypnoea and/or transient creatinine elevations [52]. Several other studies have also shown the percutaneous cryoablation of liver lesions to be safe and effective [53,54,55]. In a multi-centre randomised controlled trial, Wang et al. showed CA to be equally as safe as RFA, while achieving a significantly lower rate of LTP when compared to RFA [54].

**Figure 1:**
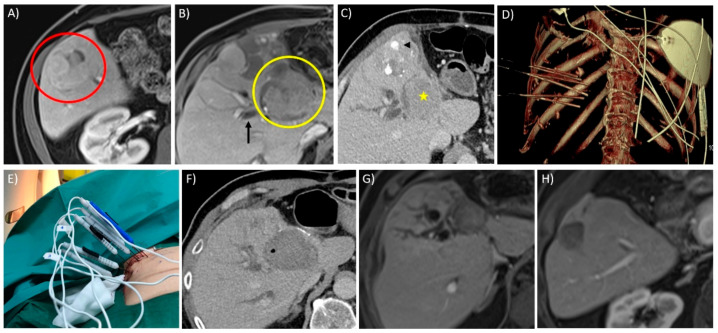
Ablation of a large, centrally located tumour using irreversible electroporation (IRE) with documented abscopal effect in a 58-year-old male with known history of hepatitis B cirrhosis and multiple prior treatments for multi-focal hepatocellular carcinoma (HCC) including trans-arterial chemoembolization (TACE), Y-90 radioembolization (RE) and trial of oral Sorafenib. (**A**) Axial T1W contrast image in the delayed phase shows a 3.8 cm HCC (red circle) in segment 5 of the liver with viable tumour despite prior trans-arterial treatments. (**B**) A more superior axial image of the same study reveals another 4.9 cm central HCC (yellow circle) with viable tumour, in segment 4 of the liver compressing the biliary confluence and causing distal biliary obstruction. With the elevated bilirubin, patient was unable to continue with oral Sorafenib and with a palliative intent, IRE was offered for this particular tumour. (**C**) Axial contrast CT image taken in the portovenous phase on the day of the procedure demonstrating enhancing viable tumour (yellow star) within the segment 4 HCC. Note the traces of lipiodol (black arrowhead) within the other tumour in segment 4, distally treated previously with TACE. (**D**) 3D volume rendering technique (VRT) image of the 7 IRE electrodes placed in order for satisfactory tumour ablation. (**E**) Photograph of the IRE electrodes over the skin during the ablation procedure. (**F**) Post-contrast axial CT image immediate post-ablation show a satisfactory ablation zone; note a tiny pocket of gas within the ablation zone, which can be commonly seen post-IRE ablation. (**G**) Axial T1W post-contrast image in the delayed phase 6 weeks after the procedure showed a complete response in the segment 4 HCC with significant reduction in the size of the tumour. Unfortunately, the biliary dilatation persisted despite the ablation, and patient’s bilirubin levels remained elevated. (**H**) Interestingly, the segment 5 tumour had also shown a complete response with reduction in tumour size, demonstrating the abscopal effect which can be seen in IRE cases.

Irreversible electroporation (IRE) is a non-thermal energy-based ablation modality that creates pores in the cellular membranes by delivering short bursts of high-voltage electrical pulses. Due to extracellular tissue architecture being preserved, IRE has an advantage over heat-based modalities near at-risk structures such as the biliary tree and vascular structures, and because it is non-thermal in nature, IRE is also not susceptible to heat sink effects and avoids incomplete ablation near major blood vessels [56,57,58,59]. IRE is shown to be safe and effective against liver tumours and is generally recommended for tumours in complex locations [60,61]. Due to its non-thermal nature, the preservation of vasculature allows the perfusion of inflammatory cells within the ablation zone. A systemic anti-tumour immune response known as the abscopal effect has been described, a phenomenon resulting in the regression of non-target lesions [62]. The downside of IRE is that it is technically challenging and time-consuming; it requires multiple (at least two) parallel electrode placements, necessitating the use of CT guidance. IRE also causes muscle contractions and has an increased risk of cardiac arrhythmia that require general anaesthesia for muscle paralysis and cardiac synchronization [63]. Another downside relates to the modality’s inability to track the ablate, resulting in an increased risk of track seeding [59]. IRE remains a promising ablation modality, although more clinical data are required to validate its efficacy. Ongoing clinical trials are evaluating its role in malignancies of the hepatobiliary tract [64,65]. Figure 1 illustrates a case of a centrally located tumour treated with IRE with abscopal effects.

High-intensity focused ultrasound (HIFU) is a heat-based ablation modality in the liver and is a non-invasive ablation modality. HIFU uses focused ultrasound at high intensities, allowing the deposition of sufficient energy to cause a well-demarcated volume of coagulation necrosis, independent of soft tissue type, first described in 1994 [66]. It has been shown to be safe and effective in the liver, either as monotherapy or in combination with other modes of treatment such as trans-arterial chemoembolization (TACE) [67,68,69,70,71,72,73,74]. The main limitation of performing HIFU in the liver is the limited window and respiratory-induced liver motion, making it time consuming and technically challenging relative to other ablative modalities, impeding its widespread adoption. HIFU has a potential role in treating patients who are poor candidates for invasive therapies such as surgery or RFA, but the exact role of HIFU in the management of liver tumours is yet to be established.

Histotripsy is a new and developing non-invasive non-heat ablation modality. While HIFU uses thermal energy to cause coagulative necrosis, histotripsy fractionates tissue using acoustic cavitation to rapidly expand and collapse bubble clouds, emulsifying the tumour tissue. Like IRE, it is inherently immune to heat-sink effects, while the risk of injury to large vessels is low due to its inherent higher mechanical strength. This new technology is still in the animal testing phase, but it has the potential to be an important ablative modality in the future [75,76]. 

## 3. Image Guidance

An accurate placement of the ablation applicator is crucial for adequate tumour ablation. Ideally, the image guidance for percutaneous liver ablation needs to detect the target lesion, enable precise applicator placement, and enable real-time or intermittent monitoring of the ablation zone.

Conventional B-mode US provides good soft tissue contrast and allows real-time targeting of the target lesion and monitoring of the ablation zone. In experienced hands, US guidance is the ideal targeting modality because it is fast, inexpensive, and does not use ionising radiation. However, US is limited by inconspicuous tumours and the limited sonographic window. As much as 45% of planned US-guided ablations are not feasible due to these factors [77]. Approximately 20–25% of small HCCs are not visible on planning preprocedural US after being detected on cross sectional imaging [78,79]. Small sizes, subphrenic locations, and the presence of liver cirrhosis are independent predictors of invisibility [78,80]. The presence of intralesional fat detected on pre-procedural MRI may predict visibility for small HCCs on US [79]. 

When the tumour is not well seen on the conventional B-mode, advanced US techniques can be employed to improve tumour visibility, these are contrast-enhanced US and CT/MR–US fusion imaging (discussed later). Contrast-enhanced US (CEUS) increases lesion conspicuousness and has been shown to improve needle placement and safety margins [81,82,83]. CEUS involves the administration of intravenous (IV) contrast agents consisting of microbubbles of gas, and has been shown to be safe and useful, even in patients with compromised renal function, unlike iodine-based contrast media [84,85]. Kupffer-phase imaging can be achieved with the IV administration of Sonazoid (GE Healthcare) due to its hydrogenated egg phosphatidylserine sodium coating, leading to persistent enhancement of the normal liver parenchyma in the post-vascular phase (which starts approximately 10 min after administration). The use of Sonazoid further increases the conspicuousness of the liver lesions and allows a longer time window for applicator placement compared with purely intravascular US contrast agents [86]. However, as of 2020, Sonazoid is only approved for clinical use in a few countries [87]. Even with intravascular contrast, US is still limited by the sonographic window, obscuration with intrapulmonary gas, and impediment by gas formation at the ablation zone.

Computed tomography (CT) guidance with CT fluoroscopy is another imaging modality that is often used for applicator placement planning and monitoring of the ablation zone. CT enables a 3D view of the tumour and surrounding structures and is better than US at monitoring the ablation zone. CT-guided ablation is useful for dome tumours which are inherently difficult to visualise on US, and may be necessary when adopting a trans-pleural approach [88]. The disadvantages include radiation exposure and a limited angle for applicator placement. Without IV contrast, soft tissue contrast is inferior to US, resulting in poor tumour visibility and suboptimal visualisation of intrahepatic vessels. With IV contrast administration, the short time-window for proper localisation and ablation of the tumour is further hampered by the non-real-time nature of the modality. 

Advanced CT techniques can be employed to mitigate the drawbacks of unenhanced CT. Intra-arterial injection of iodised oil (Lipiodol, Guerbet, Paris, France) prior to needle placement can be used to “stain” the tumour and improve visibility on unenhanced CT; Takaki et al. used this technique to perform CT-guided RFA in 150 US-invisible HCCs [89,90]. Another technique to improve tumour visibility is the use of CT arterial portography (CTAP) for hyper-vascular lesions such as HCC or hepatic arteriography (CTHA) for hypo-vascular lesions such as colorectal liver metastases. This technique improves lesion conspicuousness for applicator placement and is also useful for detecting a viable tumour seen as the incomplete-ring sign [91]. The downside of this technique is the need for an arterial puncture, which has its complications and logistically requires the procedure to be performed in a hybrid CT/angiography system. The use of stereotactic CT guidance for precise needle placement has also been used. The stereotactic image-guided navigation system consists of an optical position measurement system, retroreflective markers (placed on the patient’s skin), and an aiming device. After CT acquisition, the navigation system is used to plan the angle of the applicator trajectory as well as the depth to which the applicator is advanced. The proceduralist then positions the aiming device at the planned insertion point and aligns it with the planned trajectory, after which the applicator is inserted through the needle insert to the necessary depth. This allows accurate applicator placement that can be performed out of plane, which may improve ablation efficacy [92]. However, this requires the patient to be temporarily apnoeic during the scanning and applicator placement phases, and hence necessitates general anaesthesia (GA). Engstrand et al. described the use of high-frequency jet ventilation to limit respiratory-related liver movement to 2–3 mm, although this too requires GA and carries a higher risk of barotraumatic pneumothorax [93]. Robotic navigation systems have also been developed, which are similar to the stereotactic guidance system but use a robotic arm for targeting. The proceduralist then manually inserts the applicator through the needle guide at the end of the robotic arm to the point target [94,95].

Magnetic resonance imaging (MRI) offers superior tumour and ablation zone visibility and has the added benefit of being able to monitor ablation temperatures (thermometry) [96]. Clasen et al. found MR-guided RFA to be more effective than CT-guided RFA in a clinical trial, citing better visibility of the tumour and ablation zone as the cause [97]. MRI also offers near-real-time imaging, a higher sensitivity of small lesions, and free selection of imaging planes without the use of ionising radiation [98]. However, limited availability of MR scanners suitable for procedures, high costs, and long procedural times limit the acceptance of MRI as a mainstay guidance modality for liver ablation.

**Figure 2:**
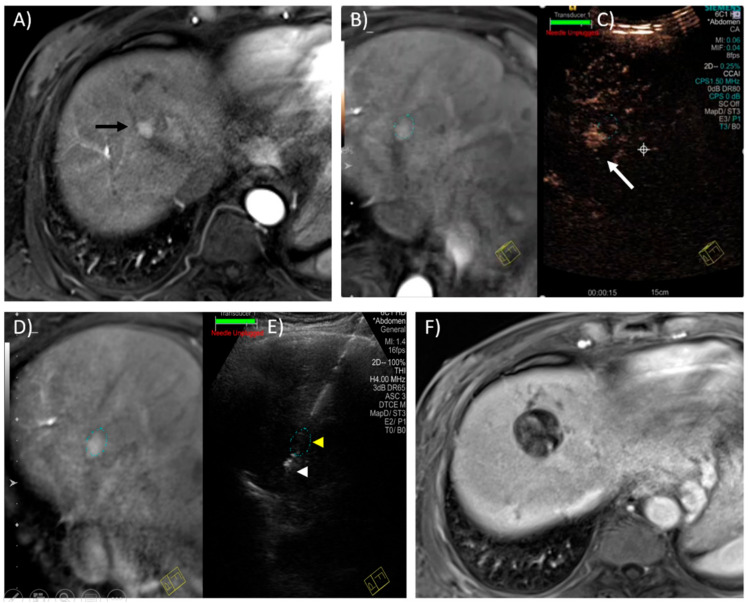
Magnetic resonance–ultrasound (MR–US)-guided fusion and contrast-enhanced ultrasound for targeting of a hepatic tumour in a 65-year-old male with known chronic hepatitis B. (**A**) Axial T1W contrast image in the arterial phase reveals a 1.4 cm, ovoid, arterially enhancing lesion compatible with HCC, in segment 8 of the liver. The lesion was radiologically occult on grey-scale ultrasound. (**B**) MR–US guided fusion performed with the patient in a slightly left lateral oblique position to improve the ultrasound acoustic window. The faint dotted oval marks the expected location of the lesion. (**C**) The lesion was not seen on grey-scale US; therefore, contrast-enhanced US was performed after administering a 2.4 mL IV bolus of Sonovue (GE Healthcare) contrast. This CEUS image captured in the arterial phase (15–45 s) reveals the enhancing lesion (white arrow) in close vicinity of the “fused” image as indicated by the faint dotted oval, thus confirming utility of the MR–US guided fusion software. (**D**,**E**) A 15 cm Emprint (Covidien) microwave antenna was placed through the expected location of the tumour (yellow arrowhead) with the tip approximately 0.5–1 cm beyond (white arrowhead). Ablation was performed in this location for 10 min at 100 W. (**F**) Axial T1W contrast image in the portovenous phase, 6 weeks later, shows a satisfactory ablation zone with no evidence of residual tumour.

Fusion imaging (FI), combining US with cross-sectional imaging, can be used to tackle the inherent limitations of each single imaging modality. CT/MR–US fusion imaging systems fuse real-time US images with CT/MR reconstruction images through an electromagnetic positioning system and 3D reconstruction data. This allows the proceduralist to target tumours only seen on cross-sectional imaging in real-time using the ultrasound probe and has been shown to be useful [99,100]. The latest guidelines of the European Association for the Study of the Liver recognise the value of CT/MR–US fusion imaging in the percutaneous ablation of liver tumours [1]. Positron emission tomography images can also be fused with US to target lesions with tracer uptake [101]. The main limitation of FI is the possibility of misregistration due to respiration and patient positioning. Figure 2 illustrates the use of MR–US FI and CEUS to aid lesion targeting.

## 4. Tumour Location and Protective/Adjunctive Measures

The location of the hepatic tumour is crucial and determines the imaging guidance used and whether protective measures are employed. Aside from bleeding and infection, many percutaneous liver ablation complications are a result of non-target injury; these include liver failure and injury to the biliary tract, hepatic vasculature, lung, gallbladder, gastrointestinal tract, and heart [102,103,104]. Adjunctive techniques can be employed after patient positioning has been optimised.

**Figure 3:**
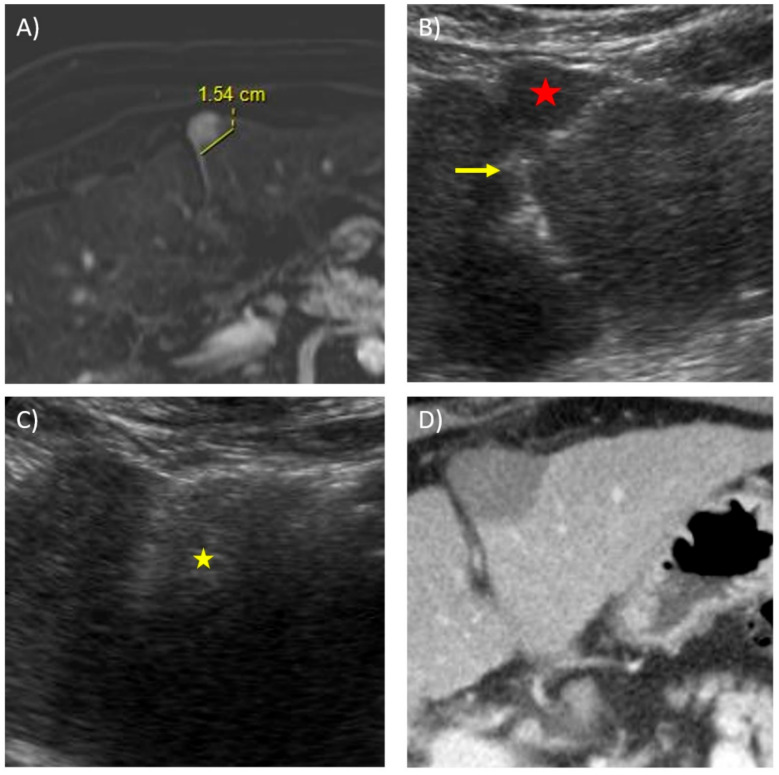
Microwave ablation of a subcapsular tumour using the “no-touch” technique in a 71-year-old male with a known history of chronic hepatitis B. (**A**) Subtracted axial T1W contrast image in the arterial phase reveals a 1.5 cm, rounded, arterially enhancing lesion in an anterior subcapsular location in segment 3, compatible with HCC. (**B**) Transverse ultrasound image at the time of ablation shows a 14 cm Soler (AngioDynamics) microwave antenna placed in a lateral-to-medial fashion posterior and slightly away from the tumour (red star), with the tip just touching the falciform ligament (yellow arrow). The aim of this no-touch technique is to create a “wedge” ablation without the antenna traversing through tumorous tissue. (**C**) Ultrasound image taken during the ablation cycle shows the echogenic storm cloud representing the ablation zone covering the lesion (yellow star). (**D**) Post-contrast axial CT image immediately post-ablation shows a satisfactory ablation zone with good margins and no residual tumour enhancement.

In general, peripheral (subcapsular) tumours can be safety ablated with similar results compared with intra-parenchymal tumours [105]. If the liver capsule appears to be involved by tumour, the capsule should be ablated as well [105]. For exophytic tumours which deform the liver border, the “no-touch” wedge ablation technique can be employed to minimise the risk of tumour seeding and bleeding [106]. Figure 3 illustrates the use of the “no-touch” wedge technique.

**Figure 4:**
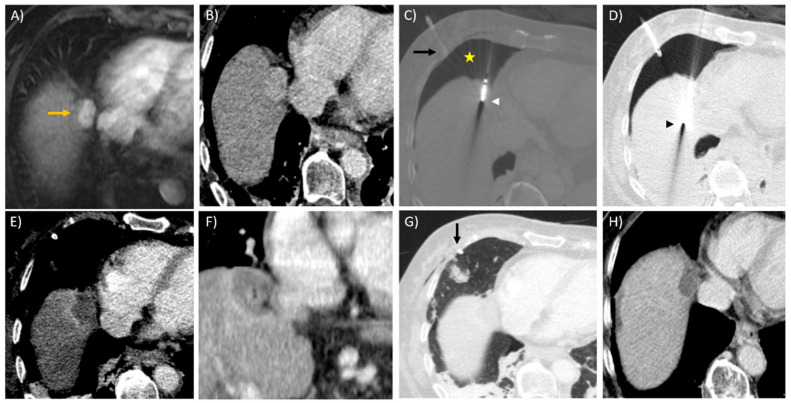
Creation of iatrogenic pneumothorax for microwave ablation of hepatic dome lesion in a 76-year-old female with known cryptogenic liver cirrhosis, and previous ablation and TACE therapies for tumours elsewhere in the liver. (**A**) Axial T1W contrast image in the arterial phase reveals a 2.1 cm, oblong, arterially enhancing lesion (orange arrow) compatible with HCC, near the hepatic dome in segment 8 of the liver. The lesion is also medially abutting the right atrial appendage of the heart. (**B**) Contrast enhanced CT image taken in the arterial phase during the time of ablation under general anaesthesia, demonstrating the tumour. (**C**) Artificial pneumothorax (yellow star) created using an 18 G cannula under CT fluoroscopy followed by subsequent insertion of an 8 F pigtail drainage catheter into the pleural space using the Seldinger technique (black arrow). A 19 cm Solero (AngioDynamics) microwave antenna was advanced intermittently under CT fluoroscopy with the tip (white arrowhead) just penetrating the diaphragmatic margin. (**D**) Further advancement of the microwave antenna with the tip seen (black arrowhead) at the posterior margin of the tumour, using the right atrial appendage as a landmark. (**E**,**F**) Post-contrast axial and coronal reconstructed CT images immediately post-ablation show a satisfactory ablation zone with adequate margins. (**G**) After the ablation, the pneumothorax is aspirated via the chest drain (black arrow) with full re-expansion of the right lung. (**H**) Axial contrast CT image taken 6 months post-ablation show involution of the ablation zone with no enhancing recurrent tumour seen.

Hepatic dome ablations are particularly challenging. Being inherently adjacent to the diaphragm anatomically, ablations close to or abutting the diaphragm can induce significant pain due to diaphragmatic irritation and increase the risk of diaphragmatic injury [107,108,109]. The creation of artificial ascites or hydro-dissection is an effective technique in minimising diaphragmatic injury. It involves the injection of fluid (usually 5% dextrose water) into the peritoneal space around the liver to separate the diaphragm from the hepatic dome, by at least 5 mm. This is often performed with a Chiba needle (14–20 G) at the level of the left subphrenic space [107]. Continued infusion of fluids may be necessary to maintain separation of the ablation zone from the diaphragm. In a retrospective review of dome lesions treated with MWA, no cases of diaphragmatic perforation or hernia were detected in both the artificial ascites and the non-artificial ascites groups [110]. However, the authors did report using lower power for the dome lesions compared with the intra-parenchymal (control) group. Another study using MWA adjacent to the diaphragm showed effective ablations with no occurrence of diaphragmatic injury when temperatures at the dome were kept at 50–60 degrees Celsius (temperature monitoring was performed using a temperature probe at the marginal tissue) [111]. In a large retrospective study of 1030 ablations, Ding et al. reported only two cases of diaphragmatic hernia (0.2%), both of which occurred with MWA, and none with RFA [34]. When access to a dome lesion using artificial ascites is not possible, the trans-pleural approach can be employed. This involves the creation of an iatrogenic pneumothorax using an 18 G epidural needle inserted into the pleural space at the anterior axillary line, separating the lung from the pleura when gas is infused. The applicator enters the diaphragm and traverses the pleural cavity (and two layers of parietal pleura) without puncturing visceral pleura or lung parenchyma [112]. After the ablation, all artificially induced pneumothoraces were aspirated fully and confirmed on a CT scan; patients were monitored with chest radiographs overnight and discharged the following day if no re-accumulation of the iatrogenic pneumothorax was detected [112]. Figure 4 illustrates the use of iatrogenic pneumothorax to access a dome lesion.

**Figure 5:**
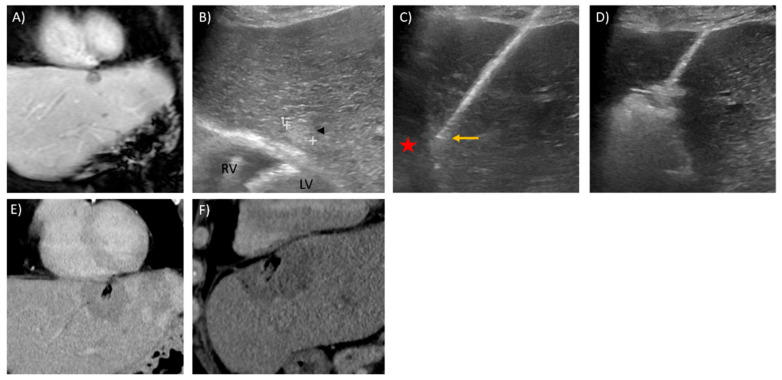
Microwave ablation of a juxta-cardiac tumour in an 80-year-old female with a known history of hepatitis B cirrhosis, Childs-Pugh A5, ECOG 0. (**A**) Post-contrast delayed coronal T1W image shows a 1.7 cm hepatocellular carcinoma (HCC) in segment 2 of the liver in a juxta-cardiac location. (**B**) Transverse-oblique ultrasound image at the time of ablation demonstrates the tumour to be well-visible, echogenic, and well-circumscribed in appearance (black arrowhead and white ‘+’). Immediately posterior to the tumour is the heart. RV, right ventricle; LV, left ventricle. (**C**) 20 cm Emprint (Covidien) microwave antenna placed under ultrasound in a sagittal orientation, with the tip guided to the centre of the tumour (yellow arrow). Antenna placement via this orientation is beneficial for 2 reasons; firstly, the heart is always visible (red star) during antenna placement to avoid inadvertent iatrogenic injury, and secondly, the ablation beyond the tip of the antenna is usually the “weakest” portion of the ablation zone, thus preventing inadvertent thermal injury to the myocardium. (**D**) Ultrasound image taken during the ablation cycle shows the echogenic storm cloud representing the ablation zone covering the lesion. (**E**,**F**) Post-contrast coronal and sagittal reconstructed CT images immediately post-ablation show a satisfactory ablation zone with no adjacent myocardial wall thickening or inadvertent iatrogenic cardiac injury.

A subset of dome tumours near the heart, known as juxta-cardiac liver tumours, defined as a tumour margin within 10 mm from the cardiac border, pose a risk of thermal injury to the heart and cardiac arrhythmias. Kwan et al. found that with precise probe placement, the thermal ablation of juxta-cardiac liver tumours can be performed safely and as effective as intraparenchymal tumours in a retrospective case series [113]. Concerning patients with implanted cardiac devices, there is a concern of interference of implanted cardiac devices when ablating close to the heart, especially because RFA uses electromagnetic currents. However, Skonieczki et al. found that there was no cardiac device interference in most RFA and all MWA cases when the device was changed to automatic pacing and the defibrillator mode was turned off [114]. Figure 5 shows a case of juxta-cardiac tumour ablation.

Ablation of non-dome peripheral lesions increases the risk of thermally induced injuries when the tumour margin is less than 10 mm from at-risk structures such as the bowel, stomach, gallbladder, or kidney. Several protective techniques can be employed to “push” these at-risk structures away from the planned ablation zone. Hydro-dissection (as described above) separates the at-risk structures from the ablation zone by infusing fluid in the plane between them. Pneumo-dissection uses the infusion of CO_2_ into the peritoneum, although large volumes of CO_2_ must be used because gas tends to diffuse from the site of infusion, when compared with artificial ascites. To maintain the ablation–nontarget organ gap, a steady flow of CO_2_ is infused, and intraperitoneal pressure of 10 mm Hg is kept for the duration of the procedure; a total of 6–8 L of CO_2_ is usually used, although less is needed for confined spaces such as the retroperitoneum [115]. The main advantage of CO_2_ over fluid is the lower thermal conductivity of CO_2_. The main disadvantage of CO_2_ is the loss of the sonographic window and the need for CT guidance.

If hydro-dissection and/or pneumo-dissection fails, the balloon interposition technique can be deployed. A 10 mm or 12 mm angioplasty balloon is placed between the planned ablation zone and non-target visceral organ over a 0.035 inch diameter stiff wire, typically Amplatz (Boston Scientific) or Rosen wire (Cook medical) after intraperitoneal access is achieved with an 18 gauge Trocar needle and secured using a 7 Fr introducer sheath. Balloon insufflation is conducted with either room air or very dilute iodine contrast [116]. Balloons offer displacement of the non-target organ by a fixed distance if well placed, although heat-induced balloon rupture can theoretically occur.

**Figure 6:**
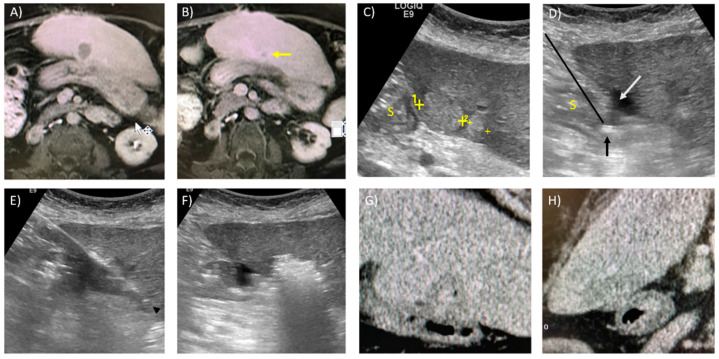
Ultrasound-guided hydrodissection with “torque” technique in a 78-year-old male with a known history of hepatitis B cirrhosis, Childs-Pugh B7. (**A**) 20 min delayed axial T1W images with Primovist contrast show a 2.1 cm hepatocellular carcinoma (HCC) in segment 3 of the liver abutting the anterior wall of the antrum of the stomach. (**B**) Slightly superior but immediately adjacent to this is a separate 0.9 cm satellite nodule (yellow arrow), also compatible with HCC. (**C**) Sagittal oblique ultrasound image at the time of ablation shows the tumours (yellow ‘+’) to be adjacent to each other, well-circumscribed, and echogenic in appearance. S, stomach. (**D**) An 18 G Chiba needle is inserted along the plane between the inferior edge of the liver and the superior margin of the stomach (black arrow) until the tip is seen beyond the stomach wall (black arrow) under real-time US guidance. D5 solution is infused through the needle, which can be seen accumulating in this space (white arrow). As more fluid is instilled, gentle force is applied in a cranial fashion on the Chiba needle externally, “torquing/levering” the stomach further inferiorly away from the liver. (**E**) A 20 cm Neuwave (Johnson and Johnson) PR XT antenna is placed through the centre of both the tumours, with the tip of the antenna (black arrow head) seen beyond the satellite nodule. (**F**) More “levering” and fluid is instilled during the ablation cycle, which is seen by the echogenic storm cloud encompassing the tumours. (**G**,**H**) Post-contrast coronal and sagittal reconstructed CT images immediately post-ablation show a satisfactory ablation zone with no adjacent stomach wall thickening, stranding or free intraperitoneal gas.

When the planned ablation zone is 5 mm away from the stomach (tumour margin 10–15 mm away from the stomach), gastric lavage can be used to protect the stomach from thermal injury. This is done by infusing chilled D5W through a nasogastric tube [117]. The same can be done for ablations close to the gallbladder. Gallbladder lavage may be performed percutaneously using a 21 gauge Chiba needle via the trans-hepatic approach, and chilled D5W is hand injected and infused after initial decompression. Lastly, applicator “torquing” is an additional technique that can be employed to displace the ablation zone away from the nontarget organ by a few more millimetres [118]. Figure 6 demonstrates the use of the torque technique.

Central tumours, on the other hand, pose a different set of problems. Large vessels, defined as vessels >3 mm, are known to cause heat sink cooling effects, increasing the risk of LTP [119,120]. The proceduralist may choose to increase power or time when performing thermal ablation close to large vessels. Placing the applicator as close as possible to large vessels can overcome the heat-sink effect [121]. The “parallel” targeting method aims to increase the contact surface between the ablation zone and the large vessel by placing the applicator parallel to the vessel wall [122]. Balloon occlusion of hepatic vasculature with peripherally inserted balloon catheters have been used to minimise the heat-sink effect posed by these large vessels [123]. Conversely, low flow in the portal vein predisposes cirrhotic patients to portal vein thrombosis [104]. Flow velocities within the inferior vena cava (IVC), hepatic arteries, and major hepatic veins are usually sufficient to prevent significant vascular thrombosis in most cases [124]. Kim et al. found 15 cases of venous thrombosis (1.1%; 12 portal and 3 hepatic veins) in a retrospective series of 1379 RFA in 1046 patients [125]. 

Thermal ablation within 1 cm of a central bile duct should be avoided to minimise the risk of biliary injury. Biliary injuries are particularly debilitating, and biliary strictures, hepatic abscess, biloma, bilioperitoneum, biliovenous fistula and biliocutaneous fistula have been reported as complications of thermal ablation [102]. The risk of thermal injury to bile ducts can be reduced by intraductal cooling measures [126]. Ogawa et al. described using an endoscopic nasobiliary drainage tube to actively perform bile duct cooling in an effort to prevent biliary injury without increasing the risk of LTP [127]. As mentioned earlier, IRE is the ideal ablative modality near these at-risk structures, as demonstrated in Figure 1 [56,57,58]. 

## 5. Follow-Up and Outcomes

Contrast-enhanced CT and MRI are the recommended modalities to assess for treatment outcome. In general, the better post-ablation imaging modality (CT or MRI) would be the modality that best evaluated the tumour on pre-ablation imaging. Both CT and MRI are good at detecting local tumour progression, new liver lesions and extrahepatic disease. MRI has superior soft tissue contrast and hence is more prone to artifacts, while CT is less prone to artifacts and can screen for lung lesions in the same setting.

Although there is no consensus on the optimal interval or frequency of post-ablation imaging, the first post-ablation imaging may be performed at 4–6 weeks after ablation to confirm complete ablation, seen as a region without enhancement larger than the treated lesion [21]. Later follow-up imaging is aimed at detecting LTP, which is much more common in the first year after ablation. After the first post-ablation imaging at 4–6 weeks, subsequent follow-up imaging can be performed at 3, 6, 9 and 12 months after treatment, and then six month intervals thereafter for at least three years [21]. Close follow-up is especially important for individuals at a high-risk of LTP, such as a poor prognostic score or suboptimal ablative margins on post-ablation imaging [128,129]. Radiomics analysis of pre-procedural imaging has been shown to aid prognostication after surgery and may play a role in predicting LTP post-ablation [130,131,132,133].

## 6. Conclusions

Percutaneous ablation is an accepted treatment modality for primary hepatocellular carcinoma (HCC) and liver metastases for non-operative candidates. The chosen ablative modality should be capable of obtaining complete tumour ablation while minimising the risk of injury to at-risk structures. Advanced techniques can be employed to improve tumour visibility on image guidance. The location of the tumour determines feasibility of ablation, the need to use adjunctive techniques to access the tumour, as well as the use of protective manoeuvres.
